# An in-depth analysis of the mitochondrial phylogenetic landscape of Cambodia

**DOI:** 10.1038/s41598-021-90145-2

**Published:** 2021-05-24

**Authors:** Anita Kloss-Brandstätter, Monika Summerer, David Horst, Basil Horst, Gertraud Streiter, Julia Raschenberger, Florian Kronenberg, Torpong Sanguansermsri, Jürgen Horst, Hansi Weissensteiner

**Affiliations:** 1 Institute of Genetic Epidemiology, Medical University of Innsbruck, Innsbruck, Austria; 2 Carinthia University of Applied Sciences, Villach, Austria; 3 Institute of Pathology, Charité - Universitätsmedizin Berlin, Berlin, Germany; 4 Department of Dermatology, Dermatopathology, Columbia University Medical Center, New York, NY USA; 5 Department of Pathology and Laboratory Medicine, Vancouver General Hospital, University of British Columbia, Vancouver, BC Canada; 6 Thalassemia Unit, University of Phayao, Phayao, Thailand; 7 Institut für Humangenetik, Universität Münster, Münster, Germany

**Keywords:** Population genetics, Evolutionary genetics, Evolutionary biology, Haplotypes, Mutation, Population genetics, Genetics, Mitochondrial genome

## Abstract

Cambodia harbours a variety of human aboriginal populations that have scarcely been studied in terms of genetic diversity of entire mitochondrial genomes. Here we present the matrilineal gene pool of 299 Cambodian refugees from three different ethnic groups (Cham, Khmer, and Khmer Loeu) deriving from 16 Cambodian districts. After establishing a DNA-saving high-throughput strategy for mitochondrial whole-genome Sanger sequencing, a HaploGrep based workflow was used for quality control, haplogroup classification and phylogenetic reconstruction. The application of diverse phylogenetic algorithms revealed an exciting picture of the genetic diversity of Cambodia, especially in relation to populations from Southeast Asia and from the whole world. A total of 224 unique haplotypes were identified, which were mostly classified under haplogroups B5a1, F1a1, or categorized as newly defined basal haplogroups or basal sub-branches of R, N and M clades. The presence of autochthonous maternal lineages could be confirmed as reported in previous studies. The exceptional homogeneity observed between and within the three investigated Cambodian ethnic groups indicates genetic isolation of the whole population. Between ethnicities, genetic barriers were not detected. The mtDNA data presented here increases the phylogenetic resolution in Cambodia significantly, thereby highlighting the need for an update of the current human mtDNA phylogeny.

## Introduction

Cambodia is located in the southern portion of the Indochina Peninsula in Southeast Asia. The Buddhist country with a population of over 15 million people (according to the General Population Census of Cambodia 2019) is bordered by Thailand to the northwest, Laos to the northeast, and Vietnam to the east. As a result of the historic expansion of the Khmer Empire in the twelfth century, the majority (96%) of Cambodia’s present-day population belongs to Khmer^1^. Indigenous minority groups of Hmong, Pong, Austronesian and Tai are collectively known as Khmer Loeu. Minority groups living in the lowlands, often among or adjacent to Khmers, include Chinese, Vietnamese and Cham. Austro-Asiatic languages are predominant in Cambodia. Being one of the most ancient language families in eastern Asia, Austro-Asiatic is also spoken in India, Bangladesh and South-western China, implying that the Austro-Asiatic speaking populations may represent the descendants of the earliest settlers of modern humans who migrated from Africa and entered into eastern Asia about 60,000 years ago^2–4^.

Genetic studies of complete mitochondrial DNA (mtDNA) have been conducted previously in populations from Southeast Asia (SEA)^5–9^, mostly in Thailand^10–12^ and Vietnam^13,14^, with very limited samples from Cambodia^2^. Besides various control region sequencing projects in populations from Southeast Asia (for example, populations from Thailand^15^, Laos^16^, Vietnam^17^, Burma^18^, and other Southeast Asian countries^19–22^), only one publication sequenced the mtDNA HVS-I (range 16,038–16,462) and HVS-II (range 65–417) from 1,054 unrelated Cambodians^2^. From these partial sequences, Zhang and colleagues analysed the complete mitochondrial genome from 98 Cambodian individuals and identified eight new and basal mitochondrial DNA haplogroups, which led to the conclusion that present-day Cambodians still carry ancient genetic polymorphisms in their maternal lineages, and most of the common Cambodian haplogroups probably originated locally before expanding to the surrounding areas during prehistory^2^.

The aim of our study was to provide a high quality mtDNA data set from Cambodia in order to understand the complex history of Southeast Asian mtDNA evolution and diversity. This was accomplished by genotyping the so far largest population set of complete mitochondrial genomes of 299 Cambodian individuals, with a cost-efficient, DNA-saving, and reliable high-throughput strategy for mitochondrial whole-genome Sanger sequencing, and by distilling ~ 7,000 mtDNA samples from studies on neighbouring- and global populations. Post-processing of the data was carried out in HaploGrep 2, estimating haplogroups and performing additional quality control steps. Phylogenetic trees were directly generated within HaploGrep 2, data exported in multiple alignment format for maximum likelihood phylogenetic tree inference with RamXL-NG^23^, Bayesian evolutionary analysis with BEAST 2^24^, Median-Joining Networks with Network.exe (v.4)^25^ as well as Maximum Parsimony trees.

## Results

The study included 299 DNA samples from Cambodian citizens, who lived in Northern Thailand at the time of sample collection. Blood samples were originally collected from 300 individuals, but one sample (C228) showed obvious signs of contamination and was therefore excluded from further analysis. Detailed descriptions of the samples including sex, age, places of birth of the sampled individuals and of their mothers, the mtDNA haplogroup affiliations and the GenBank accession numbers are given in Table S1. Our strategy enabled a 2.8-fold coverage of each mitochondrial profile with diverse electropherograms, generated with 55 forward primers and 41 reverse primers (see Supplemental Material Table S2, and Supplemental Fig. S1 for workflow). In comparison to our previously published whole-mtDNA sequencing strategy^26^, six forward primers and 20 reverse primers were new and replaced old sequencing primers. In addition, all liquid handling steps were performed by liquid handling platforms, thereby reducing potential pitfalls such as contamination or sample mix-up. Finally, the strategy was also economic in terms of DNA consumption, as less than 1 µg of DNA (for some samples only 400 ng, some had to be repeated) was needed for generating a high-quality mtDNA profile.

**Figure 1:**
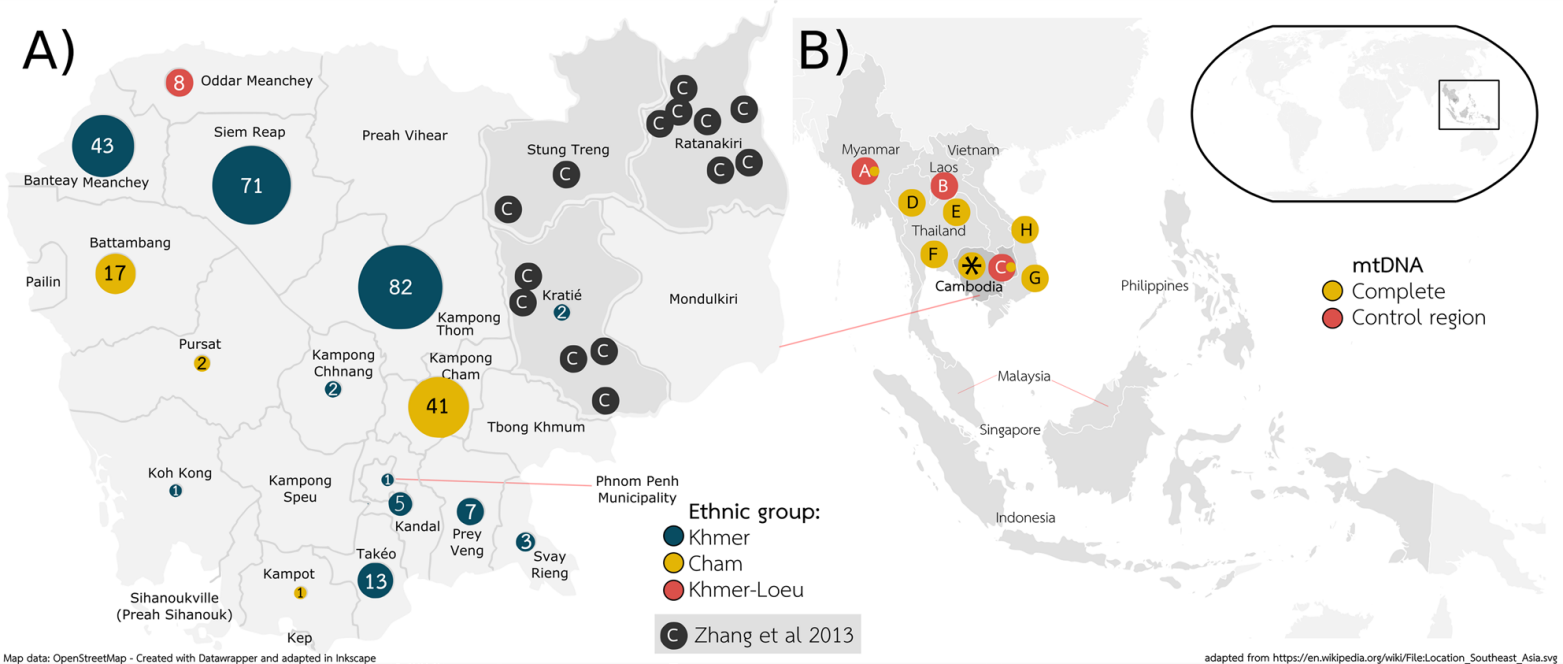
(**A**) Places of birth of samples from three different ethnic groups (Khmer, Cham, Khmer-Loeu). Additionally, the sampling locations of Zhang et al. are represented, thereby outlining the lack of Cambodian samples. (**B**) Schematic representation of data sets analysed, letters A–H representing publications with the colour denoting the completeness of the mtDNA sequences (yellow = complete, red = control region (CR) sequences). A = Summerer et al. (327 CR, 44 full)^18^, B = Bodner et al.^16^ (214 CR), C = Zhang et al. (1,054 CR, 98 full)^2^, D = Kutanan et al. (1,234 full)^10^, E = Kutanan et al. (560 full)^12^, F = Kutanan et al. (415 full)^11^, H = Duong et al. (609 full)^14^, G = 1000 Genome samples from Vietnam (KHV. 99 full). The asterisk (*) represents the present publication. Additionally the small map shows the location on the globe. (**A**) was created based on OpenStreetMap (https://www.openstreetmap.org) with Datawrapper (https://www.datawrapper.de/) and adapted in Inkscape (https://inkscape.org/), as well as (**B**) which was adapted from Wikipedia (https://en.wikipedia.org/wiki/File:Location_Southeast_Asia.svg).

Out of the 299 Samples, the majority of individuals (79.3%) were born in four provinces of Cambodia (Fig. 1, Table 1): Kampong Thom (central Cambodia, 27.4%), Siem Reap (northern Cambodia, 23.8%), Banteay Meanchey (northwestern Cambodia, 14.4%) and Kampong Cham (eastern Cambodia, 13.7%). The remaining 20.7% of samples came from twelve different provinces. In 95.3% of individuals, the places of birth of the sampled persons were identical to the maternal places of birth, thereby underscoring the stability of the geographic origin of samples (see Table 1). Figure 1 represents the place of birth of the investigated samples, the sampling locations in Zhang et al.^2^ and gives an overview of the geographic origin of samples analysed.

**Table 1:** Province of birth of sample and sample’s mothers in Cambodia.

Province of birth	Mothers Province of birth	Samples	(%)	Total samples born in Province	(%)
Banteay Meanchey	Unknown	3	7.0%		
Banteay Meanchey	Siem Reap	1	2.3%		
Banteay Meanchey	Kampong Thom	1	2.3%		
Banteay Meanchey	Battambang	2	4.7%		
**Banteay Meanchey**	**Banteay Meanchey**	36	83.7%	**43**	14.4%
Battambang	Unknown	2	11.8%		
**Battambang**	**Battambang**	15	88.2%	**17**	5.7%
**Kampong Cham**	**Kampong Cham**	41	100%	**41**	13.7%
**Kampong Chhnang**	**Kampong Chhnang**	2	100%	**2**	0.7%
**Kampong Thom**	**Kampong Thom**	82	100%	**82**	27.4%
**Kampot**	**Kampot**	1	100%	**1**	0.3%
**Kandal**	**Kandal**	5	100%	**5**	1.7%
**Koh Kong**	**Koh Kong**	1	100%	**1**	0.3%
Kratie	**Kratie**	2	100%	**2**	0.7%
**Oddar Meanchey**	**Oddar Meanchey**	8	100%	**8**	2.7%
**Phnom Penh**	**Phnom Penh**	1	100%	**1**	0.3%
**Prey Veng**	**Prey Veng**	7	100%	**7**	2.3%
Pursat	Takeo	1	50.0%		
**Pursat**	Svay Rieng	1	50.0%	**2**	0.7%
Siem Reap	Banteay Meanchey	2	2.8%		
**Siem Reap**	**Siem Reap**	69	97.2%	**71**	23.7%
**Svay Rieng**	**Svay Rieng**	3	100%	**3**	1.0%
Takeo	Unknown	1	7.7%		
**Takeo**	**Takeo**	12	92.3%	**13**	4.3%

Most of the samples lived in the same province as their mother prior leaving Cambodia (95.3%).

The largest part of participating refugees declared themselves as belonging to the ethnic group of Khmer (76.9%, Table 1 and Supplemental Table S1). However, according to the official statistics, more than 90% of Cambodians are of Khmer ethnicity^1^. The large proportion of Cham individuals sampled in this study (20.4%) could therefore be explained by the fact that the samples were collected in refugee camps in Thailand, where ethnic minorities, who were prosecuted or expelled by the Khmer rouge, found shelter.

**Figure 2:**
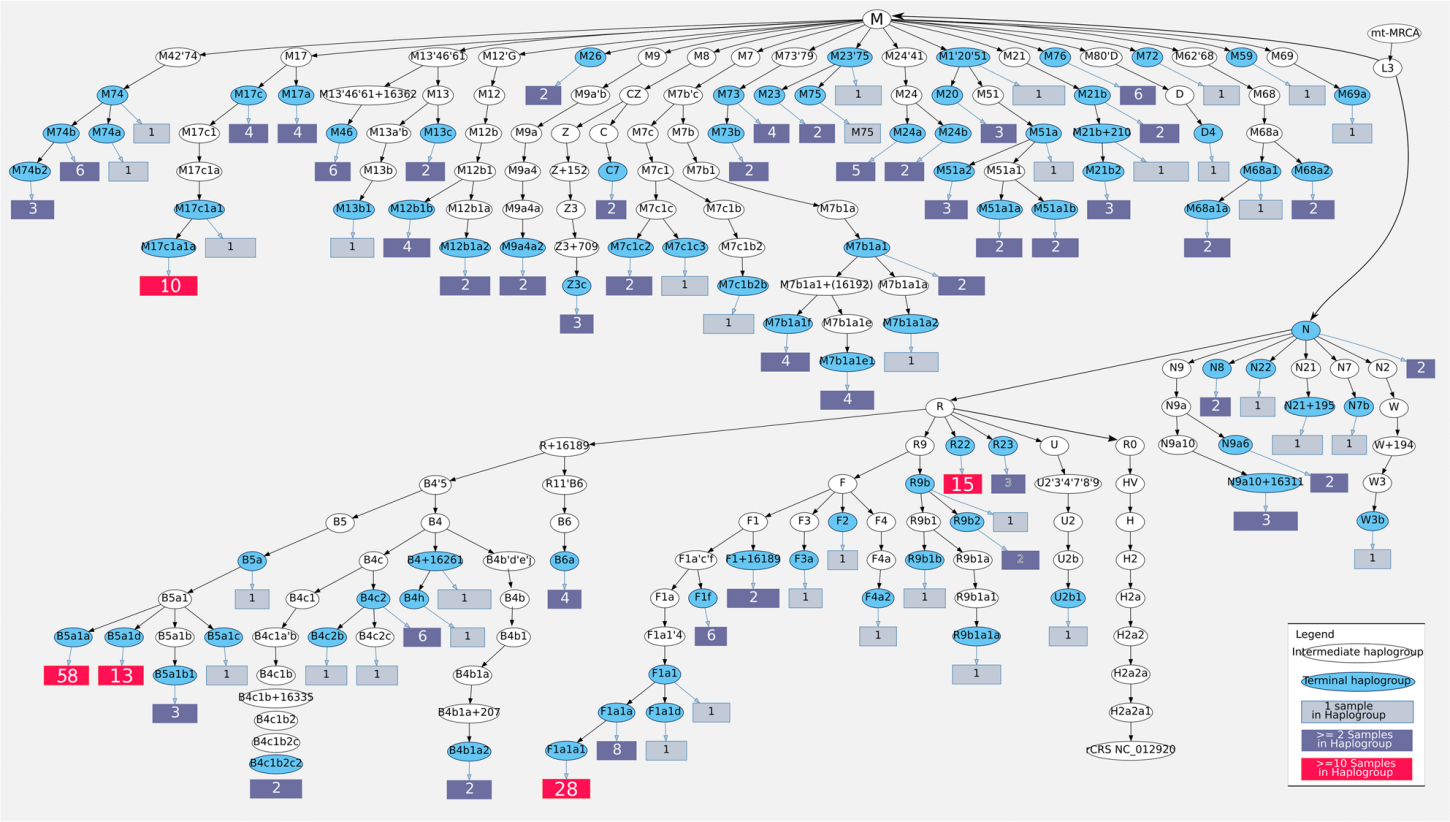
Phylogenetic representation of 299 Cambodian mtDNA samples, representing 224 unique haplotypes, classified into 90 distinct haplogroups. Haplogroups can be terminal (blue oval shaped) or intermediate nodes (white oval shaped), while rectangles indicate the numbers of samples under the corresponding haplogroup, with colour-coding according to the number of samples, as indicated in the legend at the bottom left. Figure generated with HaploGrep 2 “lineage” feature generating a DOT file, visualized with Graphviz and adapted in Inkscape.

We adapted HaploGrep 2 to automatically generate DOT graphs, which is represented in Fig. 2. The 299 samples fall into 224 unique haplotypes and into 90 different haplogroups. The samples can be grouped into macrohaplogroups R (n = 167, 55.9%), M (n = 119, 39.8%), and N (n = 13, 4.3%).

Figure 2 illustrates that macrohaplogroup M samples show only few larger clusters (with M17c1a1a being the largest clade), while 76.6% of samples in macrohaplogroup R predominantly fall into haplogroups B5a1 (75 samples), F1a1 (38 samples) and R22 (15 samples), according to the latest PhyloTree version 17^27^. This indicates a lack of phylogenetic resolution, especially in B5a1 and F1a1. Because some refugees were related to each other within the Cambodian samples presented herein, we performed the subsequent analyses by only analysing one sample per family, which left us with 264 samples (see Table S3, samples with relations marked with red-background and Table S4, where our samples are denoted by (Dataset = This)). Figures S5 and S6 represent alternative phylogenetic trees, which were generated with maximum-likelihood (RaxML-NG) and Bayesian (BEAST 2) methods respectively.

### Phylogenetic characterisation of mtDNA genomes in macrohaplogroups N and R

The 13 samples in lineage N can be classified into eight different haplogroups according to PhyloTree 17, and the 167 samples in R can be assigned to 29 distinct haplogroups (see Fig. 2). Restricting to unrelated individuals, 164 samples remained for further analysis for both N and R lineages. The samples in lineage N belong to N7, N8, N9, N21, N22, and W3b with two basal N samples.

Samples from macrohaplogroup R are mostly part of B (B4′5, B6), R9 (F1 and R9b), as well as R22 and R23 (see Fig. 2). One exception is sample C149 classified as U2b1, including several private mutations. This haplotype was observed only once in a different dataset (GenBank GQ337626, Popset 255366113), referring to an unpublished sequence, describing samples from the northeast Indo-China corridor. After removing samples with direct relationships, 60 out of 75 samples can be placed in B5a1 (20%), 32 out of 38 in F1a1 and 14 out of 15 in R22. Subsequently, we emphasized our focus on the B5a1 and F1a1 haplogroups. PhyloTree 17 lists 10 subhaplogroups to B5a1, which are grouped into the four lineages B5a1a (including B5a1a1), B5a1b (including B5a1b1), B5a1c (with B1a1c1 down to B5a1c1a1 and B1a1c2) and B5a1d. We generated maximum parsimony as well as maximum likelihood and Bayesian trees based on all previously published sequences defining B5a1 and its subgroups, by further including our B5a1 samples. Most of the Cambodian samples in B5a1 could be placed in B5a1a (77.3%), and B5a1d (17.3%), while only three samples could be classified into B5a1b and one into B5a1c. Supplemental Material Figure S2 gives an overview of the alternative trees and the extended phylogeny.

Additionally, we performed the same procedure to fine-scale haplogroup F1a1, which also includes four subgroups F1a1a to F1a1d, totalling to nine subgroups. Most samples herein could be placed in F1a1a (36 out of 38 samples, see Fig. 2). Supplemental Figure S3 gives an overview of the Bayesian phylogenetic tree, while Supplemental Table S5 provides the maximum parsimony tree for the N subset, including F1a1. While currently only two samples define haplogroup R22, our data set includes 15 samples from this lineage. Our distilled sample set for population comparison contains a total of 106 haplogroups belonging to R22, most from Cambodia (75 samples including the control region sequences from Zhang et al.^2^ comprising our data), as well as in the complete mtDNA genomes described in Thailand^10–12^, in Vietnam^13^ (only one sample out of 607 sequences), Myanmar^18^ and Laos^16^. Supplemental Table S7 gives an overview of all haplogroups identified in the data sets grouped by country. Supplemental Table S8 provides the rho ± sigma for all haplogroups for the complete mtDNA (np 1–16,569) variants and only synonymous substitutions, respectively.

### Phylogenetic characterisation of mtDNA genomes in macrohaplogroup M

Within macrohaplogroup M, we could classify all 119 samples into 48 distinct haplogroups. The M clade exhibited a larger mitochondrial diversity as the R clade. In both our and Zhang et al. Cambodian samples, comparatively few haplotypes clustered in M8 and M9 haplogroups. Haplogroups D and G, typically observed in central Asians, were underrepresented. At the same time, we detected new lineages in M20, M12b1a2, M13c, M7b1a1, M17a, M17c, M21b, M23′75, M24, and M74b. Additionally, five individuals from our study and one individual from Zhang and colleagues^2^ could be clustered into a new, deep-rooting haplogroup M46b. A group consisting of three Khmer and one Cham individuals formed the basal haplogroup M73c. Two Khmer samples from Kampong Thom and one sample from Tompoun^2^ formed the basal lineage M21b3. Four Cham samples and two Khmer samples clustered below the deep-rooting haplogroup M76. Four Khmer individuals and two Cham individuals formed the new basal lineage M74b3. Samples C140 and C199 showed an identical haplotype, although the samples were from different ethnicities. One Khmer sample from Kampong Thom and one sample of the Mon-Khmer minority Brao^2^ built the new haplogroup M74c. Supplemental Table S6 provides the maximum parsimony tree for the M subset. Additionally, median-joining networks were generated and presented in Supplemental Fig. S4.

### Relationship to populations in Southeast Asia

**Figure 3:**
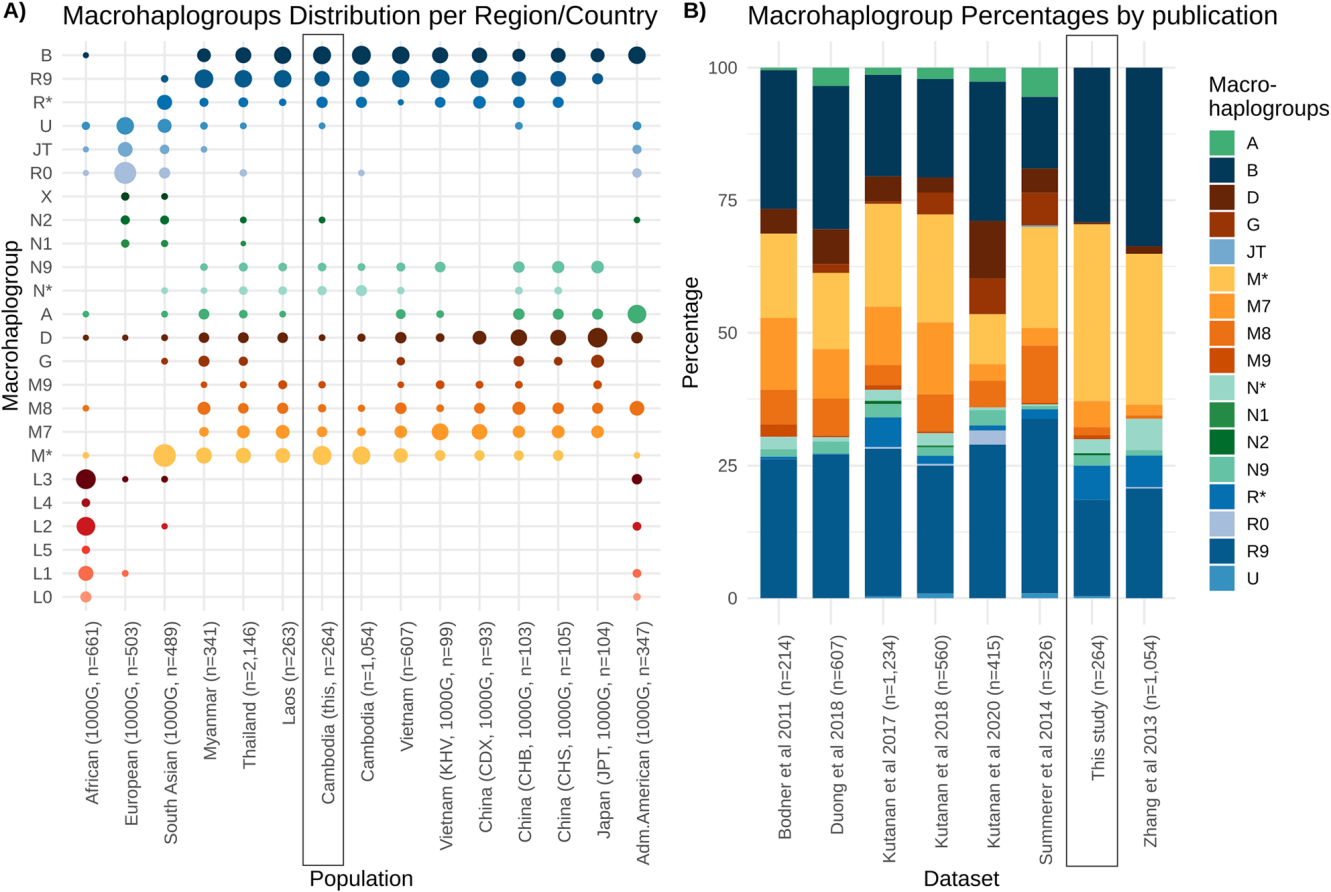
Haplogroup distribution in different populations/data sets. (**A**) Frequency plot of macrohaplogroups in different populations, including 1000 Genome Project data. The super populations are given for African ancestry, European ancestry, South Asian ancestry and admixed American ancestry. East Asia is reported individually as Vietnam (KHV = Kinh in Ho Chi Minh City, Vietnam), China (CDX = Chinese Dai in Xishuangbanna, CHB = Han Chinese in Beijing, CHS = Southern Han Chinese) and Japan (JPT = Japanese in Tokyo). (**B**) Haplogroup distribution by publication, Bodner et al.^16^ (Laos), Duong et al.^14^ (mostly Vietnam), Kutanan et al.^10–12^ (mostly Thailand), Summerer et al.^18^ (Myanmar), Zhang et al.^2^ (Cambodia). “This study” indicates the present work’s data set. Macrohaplogroups represent M (yellow), N (green) and R (blue) groups.

We compiled a data set comprising 7,178 complete mtDNA sequences as a representative data set of populations in Southeast Asia (Supplemental Table S4). The data set included 2,504 samples from the 1000 Genome Project Phase 3^28^, which we had analysed before^29^. Figures 1 and 3 give an overview of the sample locations as well as the haplogroup distributions of the different datasets. Figure 3A includes the populations from the 1000 Genome Project, with the super populations reported, (see the 1000 Genomes Project site for details https://www.internationalgenome.org/category/population/), with East Asian ancestries (EAS) are listed individually. Figure 3A highlights the observation that Cambodian samples lack the presence of haplogroup A, and exhibit only a few M7, M8 and M9 haplogroup samples compared to other Southeast Asian populations.

**Figure 4:**
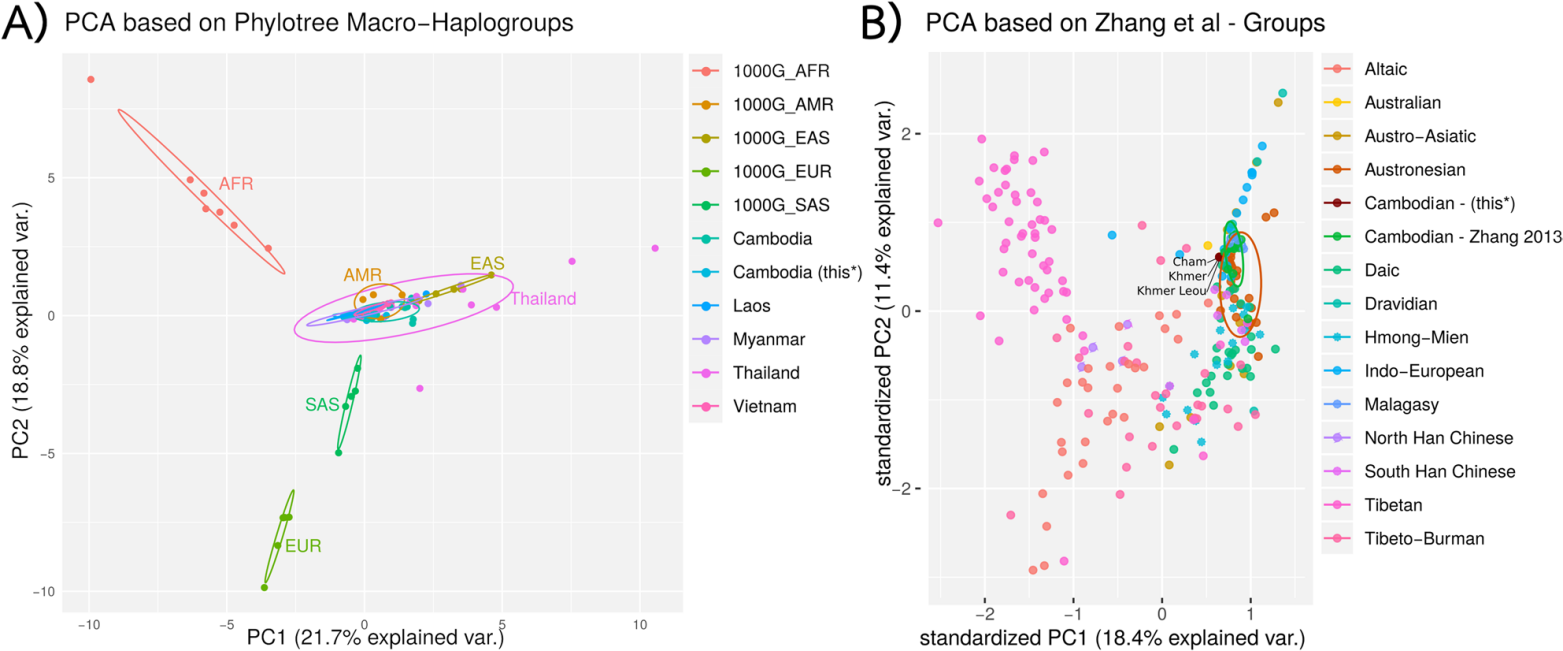
Principle component analysis (PCA) on different haplogroup levels. (**A**) PCA including global populations with the main emphasis put on populations in Southeast Asia on macrohaplogroup level. (**B**) PCA based on data from Zhang et al.^2^ with the therein described three ethnic groups (Cambodian (this*)).

Based on the HaploGrep results (see Supplemental Table S3 for detailed haplotypes), the Cambodian ethnic groups were compared to previously published mtDNA data sets from populations in Southeast Asia. For this purpose, we adapted the haplogroup classification to Zhang et al.^2^. Figure 4 shows the Principal Component Analysis plots including the global 1000 Genomes Phase 3 data. As can be seen, the first two principle components of the macrohaplogroups in Fig. 4A allow for sufficient discrimination power, with haplogroups grouped on macro-level (see Fig. 3 and Supplemental Material Table S7 for details). We observed some outliers for the Thailand samples (predominantly Northern Thailand^11^, Khon Mueang^10^, and Mon^12^) when clustering on macrohaplogroups. The close relation between Southeast Asian populations (EAS cluster) and Americans (AMR cluster) is based on the mutual presence of haplogroup lineages A, B, C, and D in both EAS and AMR super populations.

**Figure 5:**
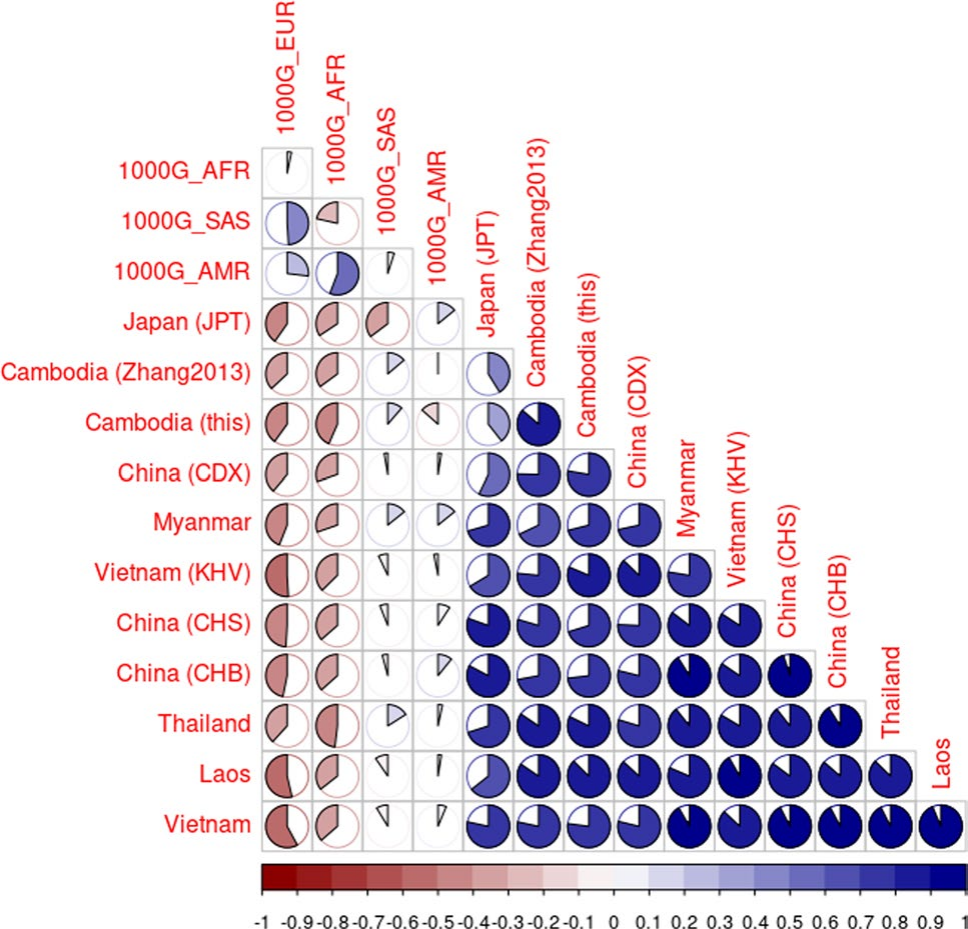
Correlation matrix (Spearman’s rank correlation coefficient) of countries on a macrohaplogroup level. Cambodia (this) represents the 264 Cambodian samples with 35 samples removed due to kinship relations. Blue colours indicate positive correlation coefficients, while red colours indicate negative correlation coefficients. The intensity of the colour and the size of the pie reflect the absolute value of the rank correlation coefficient.

In addition, we generated a correlation matrix based on the data represented in Fig. 4A. The samples for the correlation plot (Fig. 5) of the compiled dataset (n = 7,178) (Supplemental Table S4) were grouped according to the countries in Southeast Asia and on a continental level for other populations, in order to give a more compact overview. Overall, the highest correlation was found between our data set and the Cambodian samples presented by Zhang et al.^2^. The correlation plot (Fig. 5) is ordered by the first principle component, with samples from Vietnam, Thailand and Laos showing the highest correlations to each other. Interestingly, the 1000 Genome samples from Vietnam (KHV) showed a lower correlation than expected to those three neighbouring countries. Due to the lack of macrohaplogroups M7, M8, M9, and D, the Cambodian samples showed a more profound correlation to South Asians.

Additionally, we prepared the data for MitoBench^30^ (see https://github.com/mitobench/ for different repositories), which allowed the calculation of pairwise F_ST_ values based on a dataset compiled from the complete mtDNA sequences. Supplemental Material Table S9 provides the complete dataset with 5,983 multiple aligned fasta sequences, which can be directly imported into MitoBench. Two cohorts lost information, as mostly based on control region sequences, Cambodian samples from Zhang et al.^2^ (1,054 control region samples but only 98 full mt-genomes) and Myanmar samples from Summerer et al.^18^ (327 control region sequences with only 44 full mt-genomes). Supplementary Figure S7 gives an overview of all 5,983 samples, grouped by superpopulations and macrohaplogroups. Supplementary Table S10 gives the detailed matrix from the pairwise F_ST_ values, calculated with MitoBench 1.7 beta. The variation within the herein presented population was higher than between the three ethnicities (indicated by the low F_ST_ value), which however can also be the result from uneven sample sizes (Khmer (n = 201), (Cham n = 55) and Khmer Loeu (n = 8). Additionally, pairwise F_ST_ values were verified for Cham and Khmer (as Khmer Loeu had only eight samples) over all samples, showing a Weir and Cockerham mean F_ST_ estimate of 0.0027702, confirming the high homogeneity between the two groups. The populations with the lowest F_ST_ values compared to our 264 Cambodian samples were the 1,234 samples from Thailand and Laos (Kutanan et al. 2017^10^, F_ST_ = 0.0102) and the 609 Vietnamese samples (Duong et al.^14^, F_ST_ = 0.016).

## Discussion

The aim of the present study was to corroborate and extend previous phylogenetic analyses on the evolution of mitochondrial genomes in Cambodia. By whole-mitochondrial genome sequencing of 299 Cambodian individuals living in Thailand, we could define several new mtDNA haplogroups (see Supplementary Tables S5 and S6 for details).

The compilation of samples was certainly biased by the fact that the samples were not collected in mainland Cambodia, but in refugee camps close to the Cambodian border. The sample bias is reflected by the overrepresentation of individuals of Cham ethnicity in comparison to today’s ethnic composition of Cambodia. However, our careful documentation of the geographic origin of every individual, including the maternal place of birth, renders this population sample a valuable and reliable contribution for appraising the genetic diversity of aboriginal Cambodians. Our data strongly correlates to the previously published data from Zhang et al.^2^, indicating the feasibility of the present mtDNA haplotypes to better understand the genetic matrilineal makeup in Cambodia.

At present, our study provides the largest collection of entire mitochondrial genomes from Cambodia. Therewith, we were able to detect and define new mitochondrial haplogroups, predominantly under lineage B5a1 and F1a1 (see Supplementary Figs. S2 and S3 for the B5a1 and F1a1 haplogroup refinements). On this account, we emphasize the significance of further subjecting larger population samples from geographic white spots on the human evolutionary map to whole-mtDNA-sequencing projects, but also the need to update the underlying database as well as the phylogenetic representation of human mtDNA.

When considering the samples-to-haplogroup ratio, the M clade presents a lower ratio compared to the R clade. The samples-to-haplogroup ratio indicates the quotient of the number of samples divided by the number of subhaplogroups. A lower samples-to-haplogroup ratio means that there are more subhaplogroups under a specific super-haplogroup. This indicates that there are fewer pronounced clusters with many nearly identical samples. There are two explanations for this: On the one hand, the phylogeny has a higher resolution under M. This can be observed by the number of haplogroups defined in PhyloTree 17 (M includes 685 subhaplogroups at different levels), compared to R (n = 165), including B (n = 322) and F (n = 104). Nevertheless, in phylogenetic studies of Southeast Asia new deep rooting M-haplogroups with many seemingly private mutations are frequently found, indicating a still significant undersampling of macrohaplogroup M. On the other hand, the large clusters under B5a1 and F1a1 correspond to 38% of all samples, indicating that Cambodia harbours autochthonous populations with relatively recent expansions, as previously observed by Zhang and colleagues^2^. The higher number of complete sequences generated within the current project allows for a far better refinement of the haplogroups B5a1 and F1a1, extending the phylogenetic sub-clades significantly (Supplemental Figs. S2 and S3).

By generating a correlation matrix (Fig. 5) as well as F_ST_ statistics (Supplemental Table S10), we detected a bias in two studies, where both control region samples and complete mtDNA sequences were analysed. This applies to the Cambodian samples from Zhang et al.^2^ as well as our previously presented Myanmar data in Summerer et al.^18^. Supplemental Figure S7 gives an overview of the haplogroup distributions in all analysed data sets, with Supplemental Table S10 indicating the sample size per group. The vast majority of the complete mtDNA sequences fell into haplogroup M (79/98 in Zhang et al., and 33/44 in Summerer et al.), as both works focused on interesting M lineages. Therefore, it is important to consider that in both studies the complete sequences are not representative for the population structures of the two countries, and need to be handled carefully if analysed for population genetics purposes.

Our group provided the mtDNA for two larger ethnicities in Myanmar^18^, which was missing according to Zhang et al., for further evidence that Cambodia was a dispersal centre of mitochondrial lineages in east Asia^2^. The population structure in Bamar showed an extraordinarily diverse haplogroup composition. In contrast, the Karen people displayed a more profound sign of genetic isolation. While both fitted well into the Southeast Asian cluster, the two larger ethnicities in Cambodia (Cham and Khmer) showed an extremely similar haplogroup distribution, which we did not expect to this degree. As the samples derived from different regions of Cambodia, this indicates a stable gene flow between the Cambodian ethnicities, and a lower diversity than expected, compared to populations in Mainland Southeast Asia. Considering that the data derived from Cambodian refugees, it nevertheless matches the general population from different regions (see Fig. 1 for sample origins, Figs. 3 and 5 for population comparison).

Besides the phylogeographic analysis, an important aspect of this work was to ease the handling of mtDNA data. Therefore we adapted HaploGrep 2 to directly generate haplogroup overview plots such as in Fig. 2, which can be further adapted in vector graphic software (e.g. Inkscape). Thereby HaploGrep 2 provides several different lineage outputs (see https://github.com/seppinho/haplogrep-cmd for more details).

Finally, the presented dataset allows a much better fine scaling of the phylogenetic tree based on the complete mtDNA sequences. Most of the new haplogroups explored within this work are defined by variants in the coding region, with only a few new haplogroups including control region variants. The presented data also highlights the need for an update of the underlying phylogenetic tree.

## Methods

### DNA samples

Blood samples were collected from 300 Cambodian individuals, who came from 16 of the 22 regions of Cambodia (Fig. 1), but lived in Thailand at the time of the sample collection. The study was approved by the Thai Ministry of Public Health in accordance with international ethical standards. According to the Declaration of Helsinki, participation in the study was on a voluntary basis and informed consent was obtained from all donors (Chiang Mai University, Thailand, January 11, 2007). Detailed information on the geographical origin and ethnic background of the samples including the maternal region of birth was collected from all samples and can be found in Table 1 and Supplemental Table S1. Genomic DNA was extracted on a BioRobot EZ1 advanced Workstation (QIAGEN, Hilden, Germany) and quantified on an Infinite M200 NanoQuant (Tecan Group Ltd., Männedorf, Switzerland). On each plate 94 DNA samples were normalized in their DNA-concentration and arrayed on a 96-well plate with a TECAN Freedom EVO platform (Tecan Group, Männedorf, Switzerland), leaving space for two no-template controls per plate. Thereby, three full 96-well plates and one 96-well plate with the remaining DNA wells were generated. See Supplemental Material Fig. 1 with a graphical representation of the workflow.

### Amplification and post-PCR clean-up

The entire mitochondrial genome was amplified in two overlapping fragments of approximately 9 kb each^26^. For amplifying the mtDNA of one 96-well DNA plate, two 96-well plates containing PCR-mastermixes were prepared: one plate for fragment A and one plate for fragment B. Then, the DNA was transferred from the DNA-plate to the PCR-plate with a TECAN Freedom EVO platform (Tecan Group, Männedorf, Switzerland).

The DNA was amplified in a total reaction volume of 50 µl, containing approximately 280 ng (fragment A) or 120 ng (fragment B) of DNA, 1 µl of Herculase II Fusion DNA Polymerase (Stratagene, La Jolla, USA), 10 µl of 5xPCR reaction buffer, 250 µM each dNTP (Stratagene, La Jolla, USA), and 0.25 µM each primer. The PCR reaction was done in a 96-well thermal cycler (Bio-Rad Laboratories GmbH, Munich, Germany). A incubation at 95 °C for two minutes was used for initial denaturation. For a high specificity 10 touch-down cycles were applied with 95 °C for 20 s, 69 °C–0.5C°/cycle for 20 s, and 72 °C for 4 min 30 s. Subsequently, amplification was performed using 40 PCR cycles (95 °C 20 s, 64 °C 20 s and 72 °C 10 min)^26^. PCR products were purified with the QiaVac (QIAquick Multiwell PCR Purification Kit; Qiagen, Venlo, Netherlands). The post-PCR pipetting steps were performed with a Plate Mate 2 × 2 Automated Liquid Pipettor (Matrix Technologies Corp., Hudson, USA).

### Cycle sequencing, purification and electrophoresis

Each sample was sequenced with 96 primers (48 for each fragment; See Supplemental Table S2). For cycle sequencing, 1 µl of purified PCR product was combined with the sequencing master mix (containing 0.5 µl BigDye Terminator v1.1 Cycle Sequencing RR mix (Applied Biosystems, AB, Foster City, USA), 1 µl Sequencing Buffer (AB), 0.3 µM primer and distilled water up to 5 µl), and cycled (after a first denaturation step of 96 °C, 1 min) for 25 cycles of 10 s at 96 °C, 5 s at 50 °C, and 4 min at 60 °C. Cycle sequencing was performed in 384-well plates. In each 384-well plate, four different cycle-sequencing mastermixes containing four different sequencing primers were assayed into the four quadrants with the TECAN Freedom EVO platform. Then, purified PCR-products were transferred from the 96-well amplification plate to the 384-well sequencing plate with a Plate Mate 2 × 2 Automated Liquid Pipettor (Matrix Technologies Corp., Hudson, USA). In total, there were twelve 384-well sequencing plates per fragment, containing 48 different sequencing primers per fragment (Supplemental Table S2). In sum, 24 sequencing plates were cycled for obtaining full mtDNA genomes from 96 samples, after the two negative controls from PCR amplification have been replaced by PCR products from the last DNA plate (only partially filled). Cycle sequencing products were purified with MultiScreen SEQ384 Filterplates (Merck Millipore Corporation, Darmstadt, Germany). Electrophoretic separation was carried out on an ABI3730 capillary sequencer using POP-7 and a 50 cm capillary array.

### Sequence evaluation, quality assurance and data analysis

Sequence electropherograms were aligned to the revised Cambridge Reference Sequence (rCRS; NC_012920)^31^ and evaluated independently by two different mtDNA technicians with the sequence analysis software Sequencher (v5.0, GeneCodes, Ann Arbor, MI). Validation was performed by a senior mtDNA scientist using the mtDNA management software eCOMPAGT^32^. MtDNA haplotypes were assigned to haplogroups based on PhyloTree Build 17^27,33^ with HaploGrep 2^34^. In addition, the phylogenetic tree was generated directly with HaploGrep 2. HaploGrep 2 was also used for data exports in multiple alignment format, for which we also applied MAFFT on a dataset of 6,500 fasta sequences. Additionally different trees for the 264 samples (35 samples removed due to direct kinship relations) including the Reconstructed Sapiens Reference Sequence (RSRS)^35^ were generated with maximum-likelihood approaches (modeltest-ng and RAxML-NG^23^), maximum parsimony (mtphyl—https://sites.google.com/site/mtphyl/) and Bayesian evolutionary analysis (BEAUTi, BEAST 2, TreeAnnotator)^24^. The RSRS sequence was included for rooting the tree, representing the most common maternal ancestor, while we always use the rCRS for representing mutations with their according alternative nucleobase. The data was prepared for storage in MitoDB and MitoBench^30^. The latter was used for the analysis of molecular variance (AMOVA—pairwise F_ST_) in Supplemental Table S9, which was also performed on VCF files with vcftools^36^. The analysis of the data was performed in R, with libraries corrplot, corrr, ggbiplot, ggpubr and reshape2. Also the PCA was carried out in R with the standard library and the ggbiplot package. Visualization of the trees were additionally generated in the interactive Tree of Life^37^ web-application (https://itol.embl.de). Mtphyl was used for the Ro(Sigma) calculations (Supplemental Table S8) and the generation of phylogenetic trees for N and M (Supplemental Tables S5 and S6 respectively).

## Supplementary Information

Supplementary Information 1.

Supplementary Information 2.

## Data Availability

The 299 mitochondrial genome sequences reported in this study are deposited in NCBI GenBank under accession numbers KT587350- KT587648, PopSet 1020878286. The latest HaploGrep 2 version is available on https://github.com/seppinho/haplogrep-cmd
