# Supplementary material for: An in-depth analysis of the mitochondrial phylogenetic landscape of Cambodia
Source: Sci Rep. 2021 May 24;11:10816. doi: 10.1038/s41598-021-90145-2 (PMC8144189; doi:10.1038/s41598-021-90145-2)
Supplement: Supplementary file 1 — Supplementary Information 1. [file 41598_2021_90145_MOESM1_ESM.docx]

# Supplemental Material

**An in-depth analysis of the mitochondrial phylogenetic landscape of Cambodia**

The supplemental material consists of Supplementary Figures S1-S7 and legends for supplementary Tables S1-S10. The Tables are provided in the separate Excel file.

**Supplementary Figures: pages 2-8**

**Supplemetary Tables Legends: pages 9-10**

**Supplementary Tables: see seperated Excel file**


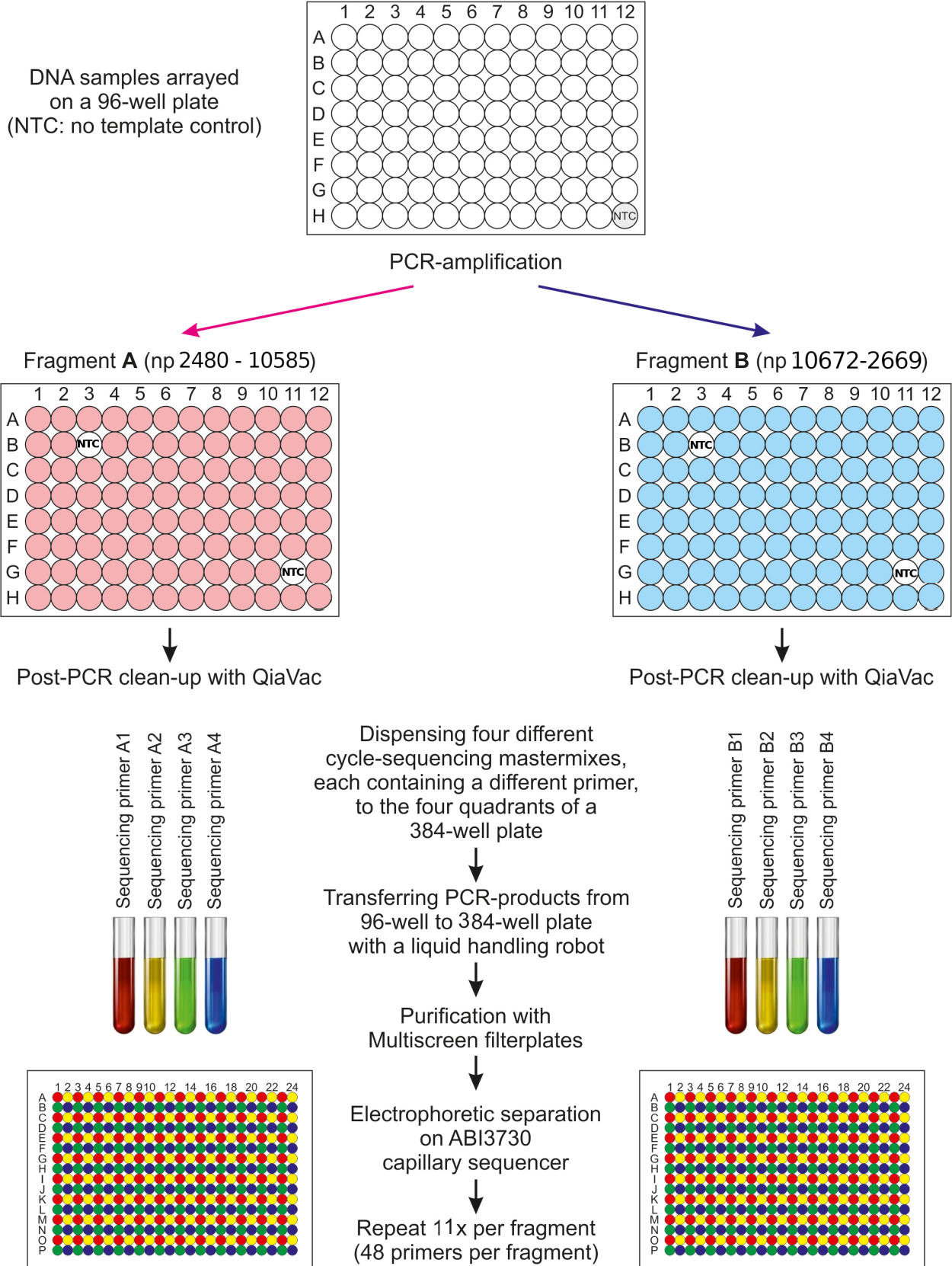

Supplementary Figure S1: Diagram representing the laboratory work flow for mtDNA whole-genome sequencing.


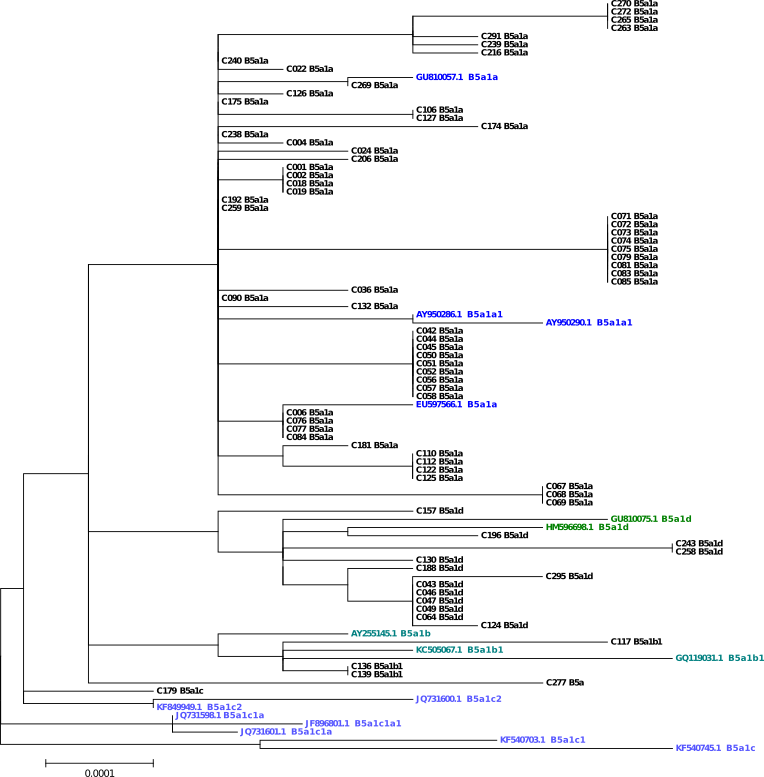


**Supplementary Figure S2**: Fine scaling Haplogroup B5a1, including all fasta sequences currently defining the subhaplogroups (B5a1a (blue), B5a1b (petrol), B5a1c (lighter blue) and B5a1d (green).


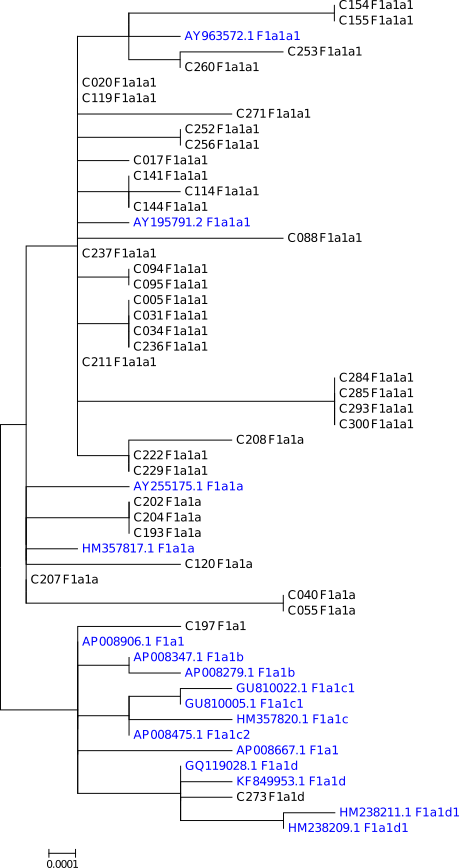


**Supplementary Figure S3**: Fine scaling Haplogroup F1a1, including all fasta sequences currently defining the subhaplogroups (F1a1a, F1a1b, F1a1c and F1a1d).


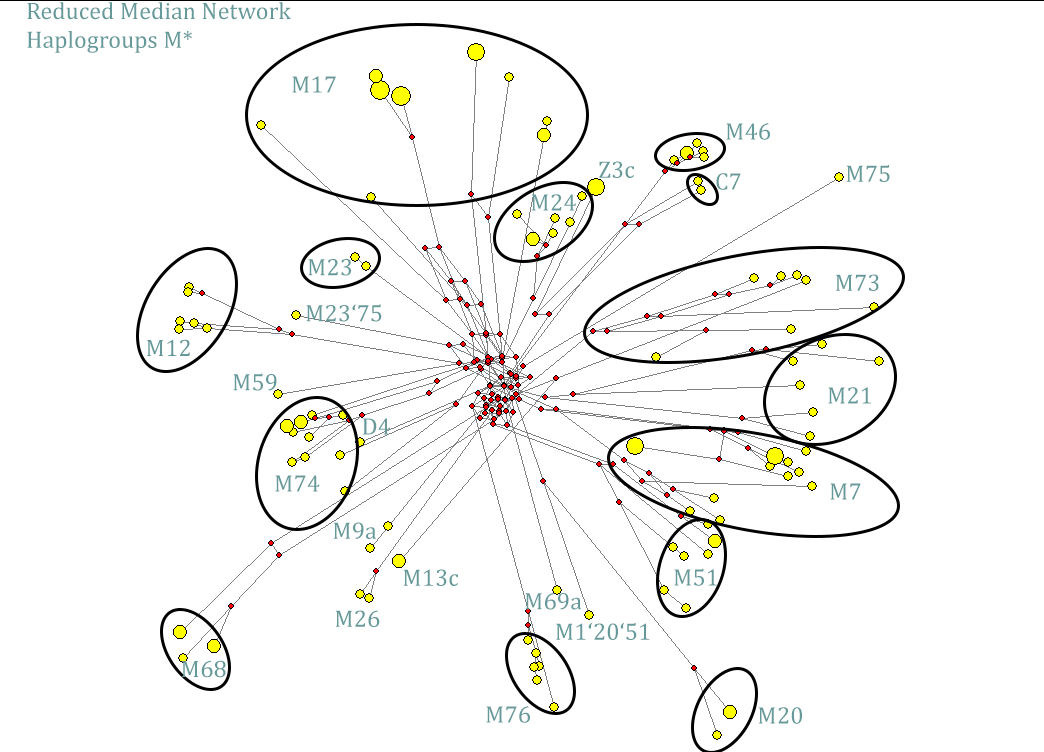

**Supplementary Figure S4**: Reduced Median-Joining Network of samples under macrohaplogroup M. Generated Network.exe 4.0, by exporting the data directly out of HaploGrep 2 in the corresponding format.


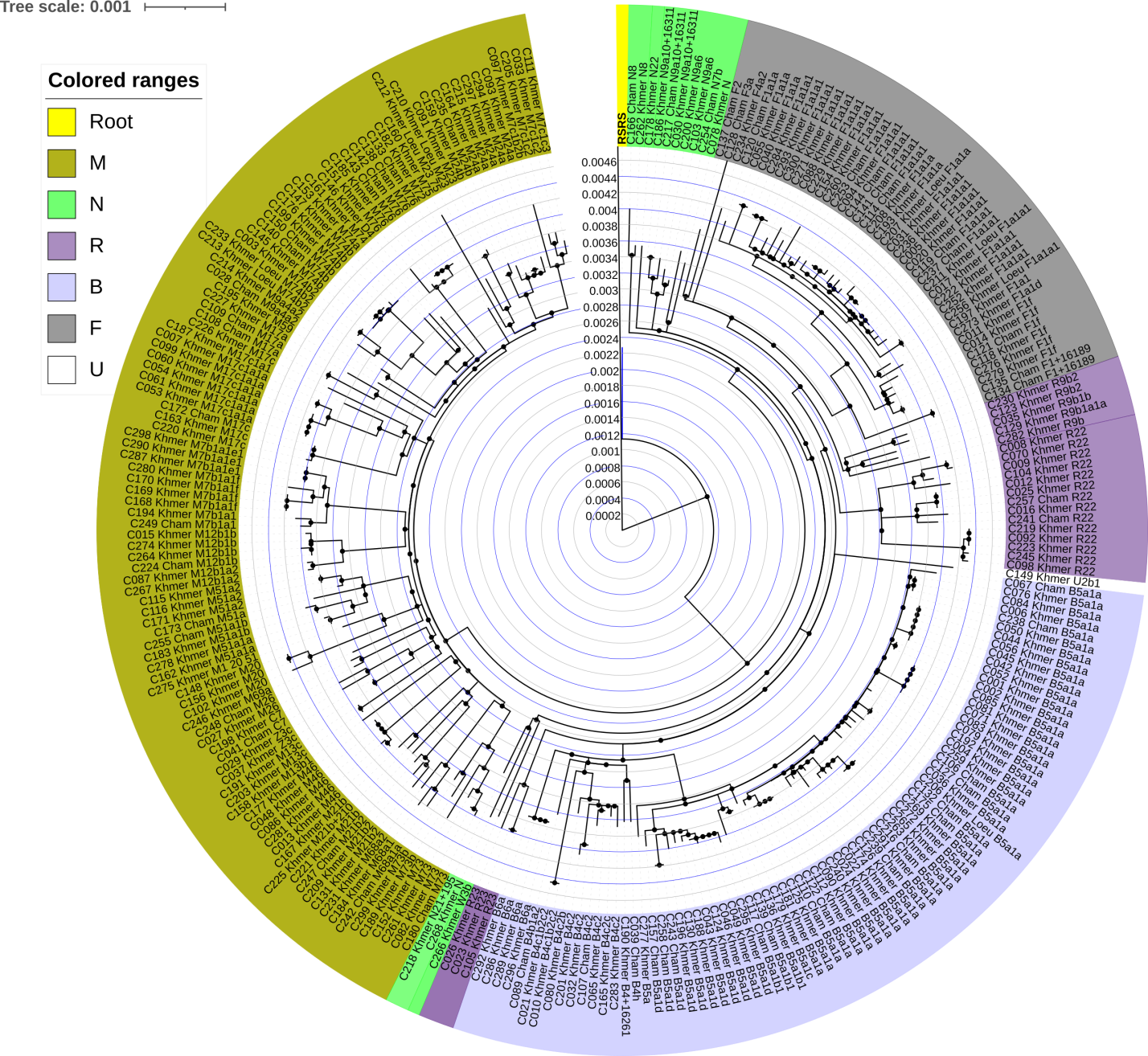


**Supplementary Figure S5**: Maximum Likelihood tree based on multiple alignments with Muscle, modeltest-ng and raxml-ng. RSRS as reference sequence. Figure generated in iTOL.


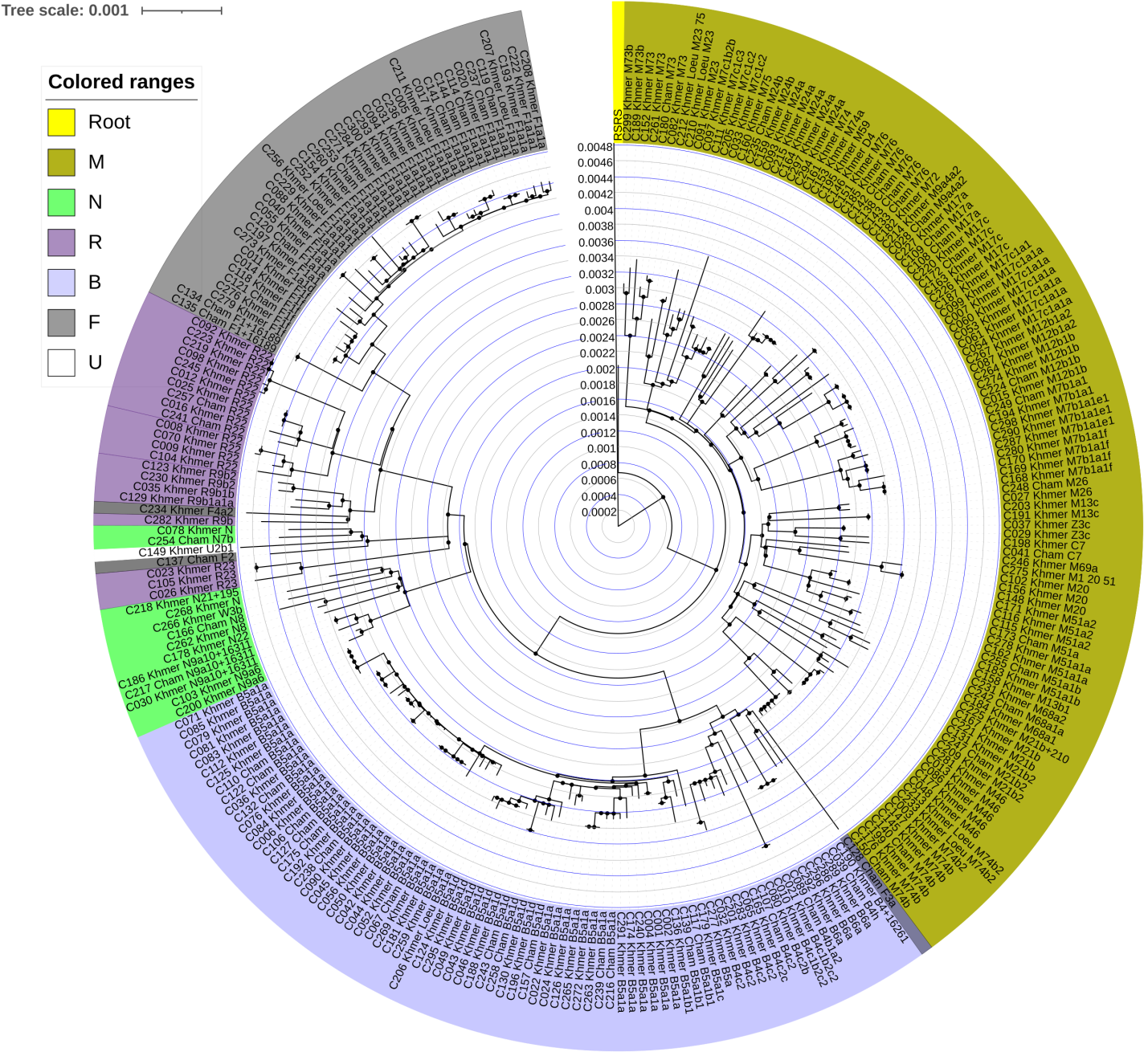


**Supplementary Figure S6**: Phylogenetic Tree from TreeAnnotator and Beast2 based on multiple alignments with Muscle. RSRS as reference sequence. Figure generated in iTOL.


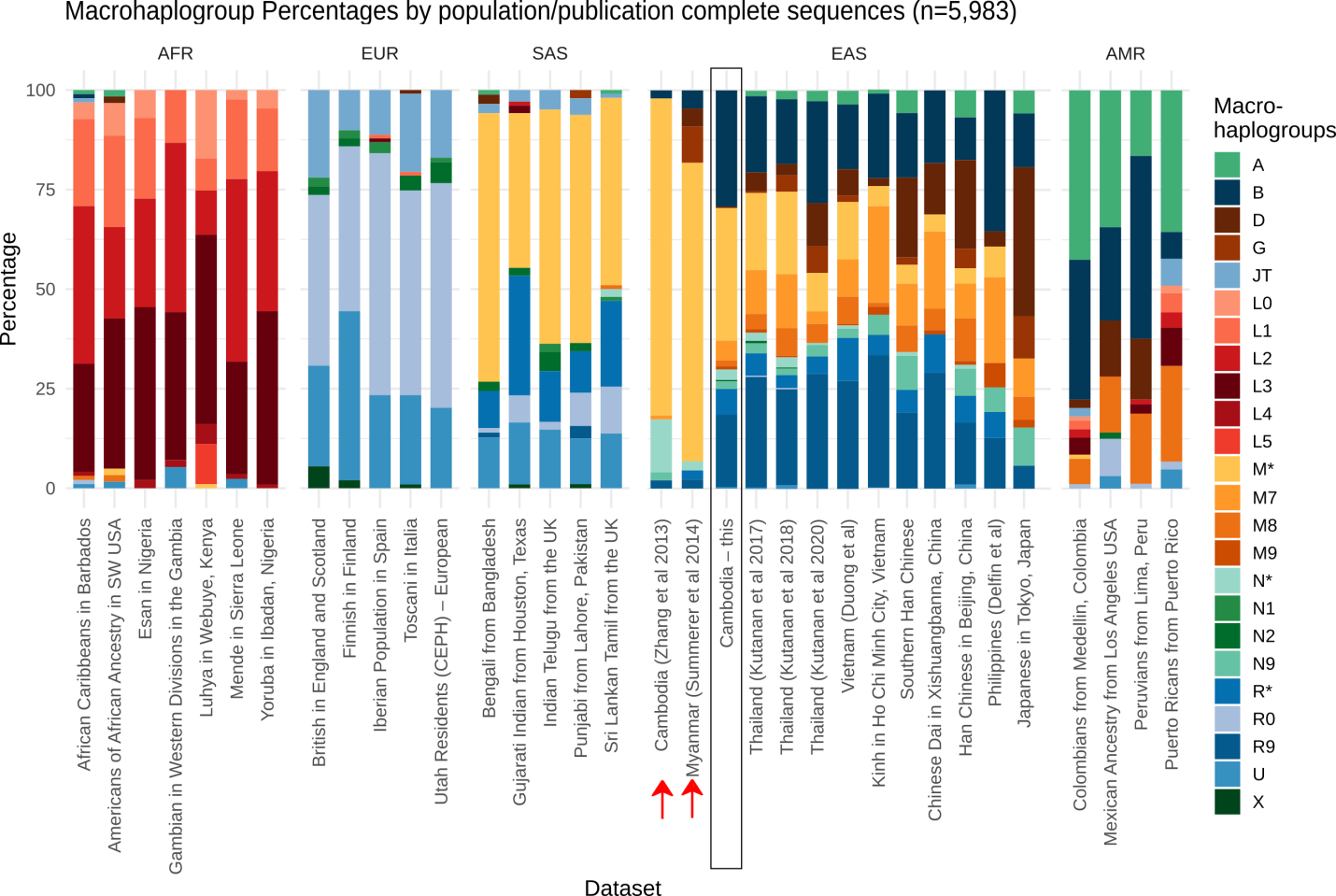


**Supplementary Figure S7**: Macrohaplogroups in different populations / publications based on complete mtDNA sequences (n=5,983). While both mtDNA population studies in Cambodian Samples from Zhang et al and Myanmar Samples from Summerer et al were performed on the mtDNA control region (see arrows indicating both sets), the complete sequences are not representative for both populations (see Figure 3 for comparison).

**Supplementary Tables:**

Supplementary Table S1 Origin, age, gender, ethnicities, haplogroup affiliations and accession numbers of mtDNA samples from Cambodia.

**Supplementary Table S2**: Primers for amplification and sequencing of the entire mitochondrial genome. Notes: Primer names indicate their direction (forward or reverse) and their 3’-position relative to the rCRS. Frag.: PCR fragment, to which the sequencing primer binds. The first four primers are amplification primers. Primers, which are updated in comparison to Kloss-Brandstätter and colleagues 2010, are highlighted in yellow.

Supplementary Table S3 HaploGrep input file, with Sample ID, the haplogroup classification result and SNPs, insertions and deletions based on the rCRS reference sequence.

Supplementary Table S4 Dataset for population comparison including a total of 7,178 samples. accession_id (Genbank ID), labsample_id (internal sample name per study), Dataset (by publication), Haplogroup_published (published haplogroup) and Haplogroup (newly assigned Haplogroup based on the FASTA sequences with HaploGrep2 and Phylotree 17). Ethnicity (published ethnic group / group abbreviation), population (consolidated populations based on ethnicity), SuperPop (super population, based on 1000 Genomes – see <https://www.internationalgenome.org/faq/which-populations-are-part-your-study/>), SuperPop2 (1000 Genomes superpopulation + countries for other datasets), SuperPop3 (1000 Genomes superopopulation for AMR, AFR, EUR, SAS and populations within EAS including country level grouping for other datasets), Supergroup (super Haplogroups with 1 letter + 1 number), FirstLetter (haplogroup first letter only except for African Lineages L0-L6), Phylotree (25 clades of the subtrees as listed on Phylotree website <http://phylotree.org/tree/index.htm>), Macrohaplogroup LMNR (macrohaplogroups grouping all L, M, N or R clades).

Supplementary Table S5 N+R macrohaplogroups maximum parsimony tree generated with mtphyl 5.003

Supplementary Table S6 M macrohaplogroups maximum parsimony tree generated with mtphyl 5.003

**Supplementary Table S7** Dataset of 7,178 mtDNA haplogroups from full and partial mtDNA sequences. Data grouped by Country (columns), and haplogroups (rows) and super-haplogroup (rows).

**Supplementary Table S8** Statistics over all samples haplogroup (n=299) coalescent timing and Ro(sigma), default parameters in mtphyl 5.003.

**Supplementary Table S9** Dataset for population comparison including a total of 5,983 complete sequences. The data can be directly imported into Mitobench v. 1.7 beta, grouped by population and subsequently used for the analysis of molecular variance.

**Supplementary Table S10** Pairwise F_ST_ calculations generated with MitoBench 1.7 beta, based on 5,983 complete sequences. The Cambodian samples within this study where analyzed twice, i.e. all 264 samples, and separated by their ethnicity (Cham (n=55), Khmer (n=201), Khmer Leou (n=8)).
